# Climate, Fertility and Oxidative Stress: Systemic and Localized Responses Associated with Ambient Heat-Induced Subfertility in Stallions

**DOI:** 10.3390/antiox15040500

**Published:** 2026-04-17

**Authors:** Narantsatsral Sandagdorj, Róisín A. Griffin, Ceilidh Jenkins, Zamira Gibb, Aleona Swegen

**Affiliations:** Centre for Reproductive Science, School of Environmental and Life Sciences, College of Engineering, Science and Environment, University of Newcastle, Callaghan, NSW 2308, Australia; roisin.griffin@newcastle.edu.au (R.A.G.); ceilidh.jenkins@uon.edu.au (C.J.); zamira.gibb@newcastle.edu.au (Z.G.); aleona.swegen@newcastle.edu.au (A.S.)

**Keywords:** heat stress, seminal plasma, blood plasma, antioxidant capacity, lipid peroxidation, oxidative DNA damage, systemic inflammation, c-reactive protein

## Abstract

Ambient heat exposure reduces male fertility in mammals with scrotal testes. Our previous work has demonstrated that some stallions are more susceptible to ambient heat-related subfertility than others, yet the mechanism for heat-induced subfertility remains uncertain, limiting both diagnosis and preventative measures. This study sought to define how the phenotype of stallions susceptible to heat-induced subfertility differs from that of more resilient animals, by measuring the systemic (blood plasma) and localized (reproductive tract) inflammatory and oxidative stress markers of sperm concentration, sperm motility assessments, total antioxidant capacity (TAC; in blood and seminal plasma), malondialdehyde (MDA; in blood and seminal plasma), oxidized guanine species (8-OH-2dG; in blood plasma and spermatozoa DNA), sperm DNA damage (assessed via Halo, SCSA (Sperm Chromatin Structure Assay) and CMA3 (Chromomycin A3)), and c-reactive protein (CRP; in blood plasma). Post-breeding dismount semen samples (*n* = 357) and blood plasma samples (*n* = 97) were collected from 31 stallions at commercial thoroughbred studs throughout one breeding season (NSW, Australia). A subset of stallions (16%) was deemed heat-induced subfertility-susceptible (HISS) stallions. These animals showed reduced seminal plasma antioxidant capacity, increased systemic and localized lipid peroxidation, and distinct systemic inflammatory response. Seminal antioxidant capacity was found to be strongly associated with impaired sperm motility (r = 0.739 * vs. r = −0.059). The plasma c-reactive protein of heat-susceptible stallions correlated to heat exposure (r = 0.597 *) and affected sperm motilities (r = −0.527 **, r = −0.434 *). Systemic oxidative DNA damage (8-OH-2dG) also increased following heat events (r = 0.862 ***) and correlated with fertility losses (FCP: r = −0.740 **, PCP: r = −0.603 *). Non-HISS stallions displayed greater variability in systemic antioxidant status and robust response following heat exposure (r = 0.307 *) and localized antioxidant capacity was more strongly correlated to systemic antioxidant capacity than in the heat-susceptible group (r = 0.897 *** vs. r = 0.482 **). We demonstrate that impaired antioxidant responses, altered redox balance and suppressed acute-phase inflammatory signalling are key features associated with heat-induced subfertility in stallions and highlight biomarkers that could be used to identify animals with heat-susceptible fertility.

## 1. Introduction

High ambient temperatures are detrimental to the fertility of male mammals [1,2,3], with concerning consequences for productivity in the face of climate change. Within species, subpopulations of animals appear to be more profoundly affected while other animals tolerate the same heat conditions with minimal fluctuations in fertility [4,5,6]. In the horse, we have previously observed a proportion of stallions that consistently exhibit subfertility following heat events, while others seem unaffected under the same commercial breeding conditions [7]. The biology underlying these distinct phenotypes is unknown. This study seeks to dissect what distinguishes susceptible animals from resilient ones, and to begin to tease out the roles of systemic and localised mechanisms in driving ambient heat-induced subfertility in stallions.

Understanding climate-related subfertility is critically important in sustaining animal production industries into the future [8]. While the effects of heat stress and ambient temperatures on fertility have been examined from several angles in livestock [8,9,10,11], this area remains scarcely studied in the horse. Horses are seasonal polyoestrous long day breeders [12] with a low fertility rate compared with other livestock [13,14]. As the breeding season of horses overlaps with spring and summer, stallions are likely to be exposed to heat conditions during critical windows of productivity [15,16]. Furthermore, the frequency, duration, and peak temperatures of heat waves have intensified in the last two decades worldwide due to climate change [17] and are expected to continue to increase in future years [18]. As such, heat-induced subfertility increasingly threatens productivity and welfare in this species, and a clear understanding of its biological drivers will be essential to alleviating these losses.

In mammals with a scrotal testis, temperatures several degrees below core body temperature are required for successful spermatogenesis, i.e., the production of fertile sperm with normal morphology. Experimental scrotal insulation or otherwise elevated testicular temperatures indeed compromise sperm production in the stallion [19], affecting germ cells and Leydig cells and impairing spermatogenesis and testosterone production [20]. Whether these mechanisms are truly relevant to real-world ambient temperature-induced subfertility is unclear. Stallions have extensive physiological adaptations to protect the testis from excessive heat and maintain homeostasis [21,22]. Whole-body heat exposure is likely to involve a more complex interplay between systemic heat stress responses and the direct effects of temperature on the reproductive tract.

Observations from our previous studies [7] indicate that the effects of ambient heat exposure under field conditions are likely to be mechanistically distinct from those induced by experimental testicular insulation. In a cohort of stallions exhibiting ambient heat-related subfertility, sperm numbers and gross sperm morphology were largely preserved, and germ cells appeared most sensitive to ambient heat events during late post-meiotic maturation and epididymal transit within the 0 to 3 weeks period prior to ejaculation. Where a decline in sperm quality was evident, it was characterised predominantly by oxidative DNA damage, supporting injury to post-meiotic and epididymal processes. We saw no evidence of impaired testicular germ cell production, which would have led to reduced sperm output and increased morphological abnormalities.

Heat-induced disruption to post-meiotic germ cell development is further supported by studies from our research centre of whole-body heat exposure in cattle and mice [23,24,25]. Collectively, these works have identified a particular sensitivity of post-meiotic germ cells—i.e., encompassing stages from round spermatids to spermatozoa transiting the epididymis—to the effects of ambient heat events. In light of these findings and our previous observations in the stallion, in the present study we focused on a two-week window of heat exposure immediately prior to ejaculation. This period captures elongated spermatids in the late acrosome and maturation phases of spermiogenesis and fully formed spermatozoa undergoing epididymal maturation and storage.

Substantial evidence also points to oxidative mechanisms driving the decline in sperm quality in heat exposed animals [26,27,28].

Along with oxidative stress, innate systemic inflammatory response mobilizes during heat stress to fight with associated damages [29,30]. C-reactive protein is one of the major acute phase proteins mobilized in response to inflammation and tissue damage, its protective anti-inflammatory mechanism activates through pro-inflammatory mediators—mainly interleukin-6—and is widely used as a non-specific, non-infection-related clinical biomarker [31,32,33]. As heat stress-induced systemic inflammation itself has a deleterious effect on male fertility [34,35], examining the interplay of oxidative damage and inflammatory response biomarkers in heat-susceptible and non-susceptible groups is crucial. Understanding this will enable us to determine why some animals are more susceptible to heat-induced subfertility and how we can better support them in a breeding setting, as well as to make better selective breeding decisions in the future.

The aim of this study was to examine associations between ambient heat exposure, fertility outcomes, and the relationship between systemic and seminal biomarkers of oxidative stress and inflammatory response in stallions with heat-susceptible fertility and heat non-susceptible fertility managed under commercial breeding conditions. Using a longitudinal, field-based dataset, we evaluated temporal relationships between climatic variables, plasma acute-phase and antioxidant measures, seminal plasma redox status, and field fertility. This work sought to identify physiological patterns that distinguish stallions exhibiting heat-associated fertility decline from those that maintain fertility under the same environmental conditions.

## 2. Materials and Methods

### 2.1. Ethics Statement

All animal handling, sampling and monitoring protocols were reviewed and approved by the Animal Care and Ethics Committee of the University of Newcastle (approval number A-2021-139).

### 2.2. Sample Collection

Post-coital semen samples (*n* = 357) and blood plasma samples (*n* = 97) were routinely collected from a cohort of commercially fertile thoroughbred stallions (*N* = 31) across four stud farms associated with the Hunter Valley Equine Research Centre, Australia over one breeding season (6 September to 10 November). Dismount semen samples were used as they provide a representative sample of the entire ejaculate [36].

### 2.3. Fertility and Climatic Data

Daily breeding data of stallions and pregnancy scan results of mares, confirmed by a reproductive veterinarian using transrectal ultrasonography 12 to 14 days after ovulation, were provided by participating stud farms and were used to calculate the fertility of weekly first-cycle pregnancy (FCP) and per-cycle pregnancy (PCP) rates for each stallion across the breeding season. Mares that returned two or more successive negative pregnancy diagnoses with no subsequent positive result were classified as ‘low-fertility mares’ and were excluded from the PCP calculations.

Retrospective climatic data were obtained from Australian Bureau of Meteorology stations located nearest to each stud farm from 18 August (14 days prior to the commencement of the breeding season) to 31 December. Data were recorded at one-minute intervals and included air temperature (°C), wet bulb temperature (°C), and relative humidity (%). From these data, maximum daily temperature (Tmax) and minimum daily temperature (Tmin) were extracted. The temperature–humidity index (THI) was calculated using the following formula [37]:

%THI = (0.8 × T_a_) + (RH × (T_a_ − 14.4)) + 46.4

where *T_a_* = air temperature (°C) and *RH* = relative humidity expressed as a proportion.

The heat load index (HLI) was calculated as follows:

HLI = 0.5 × ((T_wb_ + T_a_) − 0.31 × (RH × (T_a_ − 14.1))

where *T_wb_* = wet bulb temperature (°C).

**Figure 1:**
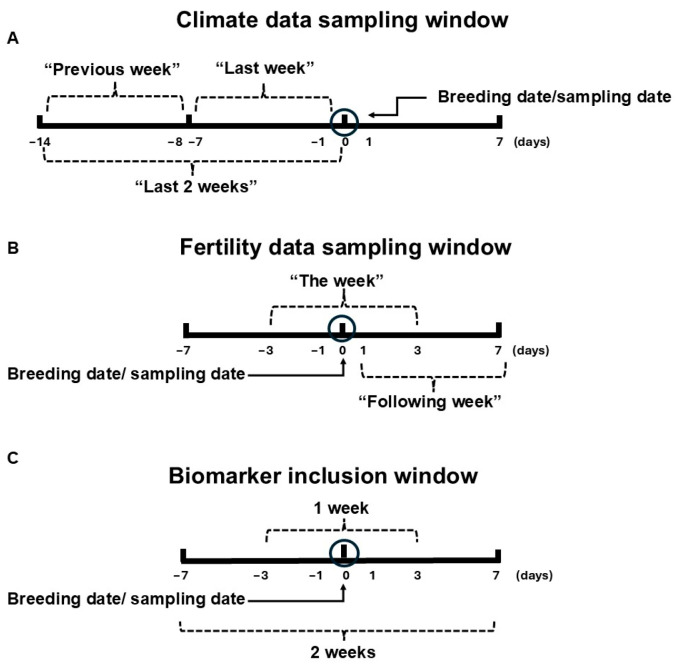
Study design: data sampling window definitions. (**A**) Climate data from meteorological stations nearest to breeding farms were obtained from BoM. (**B**) Fertility rates of stallions were given by breeding farms. (**C**) Systemic and localized oxidative stress state and some fertility biomarkers were examined by aligning climate and fertility data.

From these data, the peak Tmax, Tmin, THI and HLI were calculated for the periods preceding the sampling date as per Figure 1.

### 2.4. Finding Stallions with Ambient Heat Susceptible Fertility and Their Susceptibility Time Window

Fertility rates of FCP and PCP of the week (day −3 to 3) and following week (day 1 to 7) were correlated with peak climate indices—maximum temperature (Tmax), minimum temperature (Tmin), heat load index (HLI), and temperature–humidity index (THI)—from three time windows—last week (day −1 to −7), previous week (day −8 to −14), and the combined last two weeks period (day −1 to −14)—as shown in Figure 1.

Stallions showing significant negative correlations (*r* ≥ −0.2, *p* ≤ 0.05) in at least two combinations of any fertility measure and climate index were classified as stallions with heat-susceptible fertility. Stallions whose fertility did not display negative associations with climate indices (*p* ≥ 0.1) were classified as heat non-susceptible fertility.

### 2.5. Reagents

Unless specified, all research grade chemical reagents were obtained from Sigma-Aldrich (Castle Hill, NSW, Australia). A modified Biggers, Whitten and Whittingham medium [38] was used to prepare and incubate spermatozoa for analyses, and contained 95 mM NaCl, 4.7 mM KCl, 1.7 mM CaCl_2_·2H_2_O, 1.2 mM KH_2_PO_4_, 1.2 mM MgSO_4_·7H_2_O, 25 mM NaHCO_3_, 5.6 mM D-glucose, 275 mM sodium pyruvate, 3.7 μL/mL 60% sodium lactate syrup, 50 U/mL penicillin, 50 mg/mL streptomycin, 20 mM HEPES (GE Healthcare, Parramatta, NSW, Australia) and 0.1% (*w*/*v*) polyvinyl alcohol, with an osmolarity of 310 mOsm/kg and a pH of approximately 7.2.

### 2.6. Sample Processing

Semen collection required residual semen to be gently milked from the urethra as the stallion dismounted the mare, which was collected directly into a prewarmed sterile specimen container. One part of raw semen was centrifuged for 3 min (400× *g*), and top layer seminal plasma was collected and centrifuged again for 10 min (5000× *g*) to remove residual cells. Seminal plasma was then aspirated into a fresh tube and stored at −80 °C for future analyses. To isolate high-quality spermatozoa from other cell types, debris and seminal plasma, raw semen was diluted (1:1) with pre-warmed EquiPlus extender (Minitube Australia, Ballarat, Australia) and processed via single-layer colloidal centrifugation on-site using EquiPure gradients (Tek-Event, Round Corner, NSW, Australia; 400× *g*, 20 min). Sperm pellets were resuspended in BWW and maintained at room temperature (RT) for motility and concentration analyses. Isolated spermatozoa in BWW were snap-frozen in liquid nitrogen and stored at −80 °C until analysed.

Blood samples were collected by staff personnel from the same cohort of stallions, during the breeding season (6 September to 10 November), into EDTA coated vacutainers (BD, Macquarie Park, NSW, Australia) and immediately cooled to 4 °C. Within one hour of collection, samples were then centrifuged for 10 min (1000× *g*) at room temperature. Blood plasma was harvested and stored at −80 °C for future analyses.

### 2.7. Sperm Concentration

Sperm concentrations were determined using a NucleoCounter NC-100™ (ChemoMetec, Allerod, Denmark). Samples of 5 µL of semen were diluted with 1 mL S100 dilution buffer, gently mixed and vortexed 3 times for 5 s each. The mix was aspirated through dipping SP1 cassette using its piston and read by the machine.

### 2.8. Motility Analysis

Sperm motility was objectively determined on-farm using an iSperm device (version 4.5.2; Aidmics Biotechnology Co., Ltd., Taipei City, Taiwan) according to manufacturer’s instructions. Each sample was assessed in triplicate, using the supplied base and cover chips. Total motility (%), progressive motility (%), average path velocity (VAP, μM/s), curvilinear velocity (VCL, μM/s), straight-line velocity (VSL, μM/s), straightness (STR), and linearity were assessed. Cells exhibiting VAP ≥ 20 μM/s and VSL ≥ 3 μM/s were considered motile (total motility). Cells exhibiting VAP ≥ 50 μM/s and STR ≥ 75% were considered progressive.

### 2.9. Sperm DNA Integrity

#### 2.9.1. Oxidized Guanine Species (8-OH-2dG)

Sperm DNA was isolated by magnetic bead extraction method using an sbeadex Livestock Purification Kit (NAP44701; Biosearch Technologies, Berlin, Germany) and DNA concentration and purity were measured using Qubit 1x dsDNA High Sensitivity Assay Kit (Q33230; Thermo Fisher Scientific, Waltham, MA, USA), according to the manufacturer’s instructions. Isolated genomic DNA was then subjected to two cycles of heat treatment consisting of incubation at 100 °C for 3 min, followed by freezing at −80 °C for 5 min. Samples were hydrolysed using nuclease P1 enzyme (0.1 U/1 µg of DNA) in an ammonium acetate buffer (50 mM NH_4_CH_3_CO_2_, 1 mM ZnCl_2_, pH 6.0) and incubated for 2 h at 56 °C. Samples were then incubated in (0.001 U/1 µg of DNA) shrimp alkaline phosphatase in 1 M Tris (pH 9.0), contained at 37 °C for 30 min, before being heated to 100 °C for 10 min. Total 8-OH-2dG was assessed with the DNA/RNA Oxidative Damage (High Sensitivity) ELISA Kit (589320, Cayman Chemical, Ann Arbor, MI, USA) according to the manufacturer’s protocol and using 1 µg of sperm DNA per assessment. Absorbance was measured at 415 nm using the microplate reader (SpectroStar Nano, BMG Labtech, Ortenberg, , Germany).

#### 2.9.2. Sperm Chromatin Dispersion Assay (Halo Assay)

The presence of single strand breaks in sperm DNA was assessed using the sperm chromatin dispersion (‘Halo’) assay, as previously described [39]. Briefly, spermatozoa were snap-frozen on-site in liquid nitrogen, before being stored at −80 °C for analysis. At the time of assessment, snap-frozen sperm cells were thawed over ice, and 100 μL was mixed with 1.0% low melting agarose (Bio-Strategy, Tingalpa QLD, Australia; 7:3, agarose:sperm). A 70 µL aliquot of the sperm–agarose solution was spread evenly across a prewarmed (35 °C) microscope slide, pre-coated with 0.65% agarose. Slides were sealed with a coverslip and incubated at 4 °C for 10 min. Coverslips were removed and the slides were treated with HCl (0.08 N) for 7 min (RT), followed by ‘Halo solution 1’ (0.4 M Tris, 1% SDS, 50 mM EDTA, 0.8 M DTT, pH 7.5) for 20 min (RT), and ‘Halo solution 2’ (0.4 M Tris, 1% SDS, 2 M NaCl, pH 7.5) for 5 min (RT) to lyse the cells and to relax and neutralise the DNA. Slides were then treated with Tris–boric acid–EDTA buffer (TBE; 0.1 M tris, 0.09 M boric acid, 0.002 M EDTA, pH 8.0) for 2 min (RT), before washing with increasing concentrations of ethanol (70%, 90%, and 100%) for 2 min each (RT). Slides were airdried for 15 min, before counterstaining with the nuclear marker, 4′,6-diamindino-2-phenylindole (DAPI; 1:2000 in PBS), for 2 min (RT). Slides were then rinsed with PBS (×2) to remove unbound stain. Mowiol mounting medium was placed on the slides before sealing with a coverslip. Slides were visualised using an AXIO Imager.A1 fluorescence microscope (Carl Zeiss Micro Imaging GmbH, Jena, Thuringia, Germany). At least 100 cells per sample were evaluated by an assessor blinded to sample identity. Spermatozoa were categorised based on the presence and size of halos surrounding the nucleoid. Cells with halos approximately three times the diameter of the inner nucleoid were classified as ‘large’, those with limited DNA extension beyond the nucleoid as ‘small’, and those with no visible halo as ‘no halo’. Cells with large halos were interpreted as having intact DNA, whereas sperm with small or no halos were considered to exhibit DNA fragmentation.

#### 2.9.3. Sperm Chromatin Structure Assay (SCSA)

SCSA was performed using a BD FACSCanto II flow cytometer (BD Biosciences, San Jose, CA, USA) operated with FACSDiva software (v9.0.1). A 488 nm argon laser was used with a 530/30 nm band-pass filter to detect green fluorescence (intact double-stranded DNA) and a 670/30 nm band-pass filter to detect red fluorescence (denatured single-stranded DNA). DNA fragmentation was expressed as the percentage of spermatozoa outside the main population (%DFI), as previously described [40]. Spermatozoa were snap-frozen on-site in liquid nitrogen, before being stored at −80 °C for analysis. At the time of assessment, snap-frozen sperm cells were thawed over ice. A 100 µL aliquot of sperm suspension (~10 × 10^6^ sperm/mL) was transferred into a FACS tube and incubated with 200 µL of acid detergent solution (0.8 N HCl, 0.15 M NaCl, 0.1% Triton X-100; pH 1.2) for 30 s at RT. Following incubation, 600 µL of acridine orange staining solution (0.1 M citric acid, 0.2 M Na_2_PO_4_, 1 mM EDTA, 0.15 M NaCl, 1 M acridine orange) was added, and the cell solution was equilibrated for 2.5 min before being immediately analysed via flow cytometry. A total of 10,000 events were recorded per sample, while no halos were considered to exhibit DNA fragmentation.

#### 2.9.4. Chromomycin A3 (CMA3)

Spermatozoa were fixed in 2% paraformaldehyde for 10 min at 4 °C on-site, washed in phosphate-buffered saline (PBS), and stored in 0.1 M glycine in PBS until analysis. An aliquot (50 µL) of the fixed sperm suspension was placed onto poly-L-lysine-coated coverslips and allowed to settle for a minimum of 4 h at 4 °C. Cells were then permeabilised at room temperature (RT) for 15 min in 0.2% Triton X-100 prepared in PBS, followed by washing with McIlvaine’s buffer (10 mM MgCl_2_, 82.35 mL 0.2 M Na_2_HPO_4_·12H_2_O, 17.65 mL 0.1 M citric acid). After removing excess buffer from the coverslips, 25 µL of the chromomycin A3 (CMA3) working solution (1:40 dilution of CMA3 in McIlvaine’s buffer) was applied, and the coverslips were incubated for 20 min at RT. Coverslips were subsequently washed with McIlvaine’s buffer and mounted on microscope slides with 10 μL of Mowiol per coverslip. Slides were visualised by fluorescence microscopy (Axio Observer, Zeiss, Jena, Germany) using an excitation/emission maximum of 445 nm/575 nm. A total of 50 sperm cells were evaluated per sample. Cells exhibiting bright CMA3 fluorescence were classified as CMA3 positive, whereas those showing faint or ambiguous staining were classified as CMA3 negative.

### 2.10. Systemic and Localized Biomarkers

To characterise the systemic inflammatory and oxidative stress responses associated with heat-induced subfertility, sperm quality parameters (concentration, motility, and DNA integrity), seminal plasma redox markers (total antioxidant capacity and malondialdehyde concentration), and systemic biomarkers of redox and inflammatory status (total antioxidant capacity, malondialdehyde, c-reactive protein concentration, and oxidative DNA damage) were compared between susceptible and non-susceptible stallions, using samples collected throughout the breeding season. Interactions between systemic and seminal markers were assessed to determine how systemic physiology may influence the local reproductive environment in susceptible versus non-susceptible stallions. All colorimetric and absorbance assessments were conducted using microplate reader (SpectroStar Nano, BMG Labtech, Ortenberg, BW, Germany).

#### 2.10.1. Total Antioxidant Capacity (TAC)

The TACs of blood plasma and seminal plasma were measured using the Antioxidant assay kit (709001, Cayman chemical, Ann Arbor, MI, USA) according to the manufacturer’s instructions. Samples were thawed on ice and diluted either 1:10 (seminal plasma) or 1:15 (blood plasma) with Milli-Q water (Millipore, North Ryde, NSW, Australia) prior to analysis.

#### 2.10.2. Malondialdehyde (MDA)

Lipid peroxidation end-product MDA was examined on undiluted seminal and blood plasma samples using a commercial colorimetric kit (ab233471; Abcam, Cambridge, UK), according to the manufacturer’s instructions.

#### 2.10.3. C-Reactive Protein (CRP)

Blood plasma CRP was assessed using a Horse CRP ELISA kit (190527; Abcam, Cambridge, UK) in accordance with the manufacturer’s instructions. Plasma was diluted 100 times with Milli-Q water (Millipore, North Ryde, NSW, Australia) prior to analysis.

### 2.11. Statistical Analysis

Multivariate correlations were performed to identify heat-susceptible stallions using JMP Pro 18 (version 19.0.5, SAS institute Inc., Cary, NC, USA) with 95% confidence intervals and a significance threshold of *p* ≤ 0.05, whereas non-susceptible stallions had a threshold of (*p* ≥ 0.1). A non-parametric Mann–Whitney U test was used on comparison between groups (*p* ≤ 0.05) and the results are presented as mean ± SEM. Pearson correlation coefficients with 95% confidence intervals were used to evaluate relationships among seminal and systemic biomarkers by GraphPad Prism (version 11.0.0.(84) GraphPad STANDARD Inc., Solana Beach, CA, USA). Significance was set at *p* < 0.05, denoted as (*); *p* < 0.01, denoted as (**); *p* < 0.001, denoted as (***); and *p* < 0.0001, denoted as (****), indicating significant, highly significant, very significant, and extremely significant differences between the susceptible and non-susceptible groups, respectively. Coefficients of determination (R^2^) were reported to describe the proportion of variance explained.

## 3. Results

### 3.1. Classification of Susceptible and Non-Susceptible Stallions

**Figure 2:**
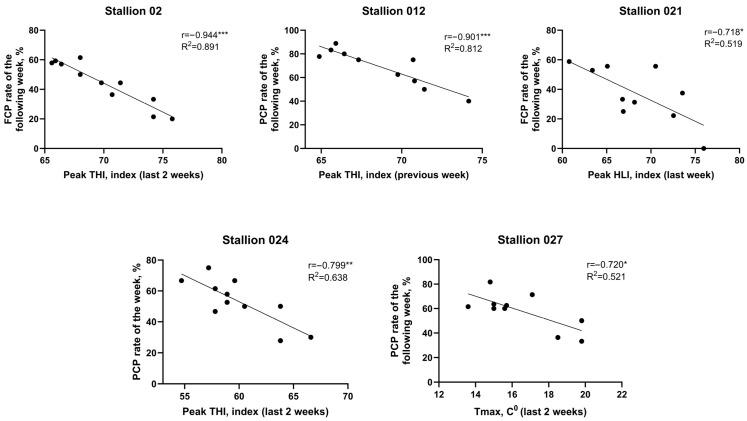
Fertility rates of some stallions had very strong negative correlation to climate indices. Multiple strong negative correlations between fertility rates and the climate indices of different heat periods were detected in some stallions (highest negative correlations per stallion are shown). PCP: per cycle pregnancy; FCP: first cycle pregnancy; THI: temperature–humidity index; HLI: heat load index; Tmax: daytime peak temperature. *, *p* < 0.05; **, *p* < 0.01; ***, *p* < 0.001.

Of 31 stallions analysed, five (16.1%) displayed significant negative correlations between heat variables and fertility rates; these stallions were deemed heat-induced subfertility susceptible (HISS) stallions. Correlation values ranged from r = −0.72 * to r = −0.944 *** (Figure 2).

More correlations were observed between fertility and climate indices across the window of the final 2 weeks preceding sampling (*n* = 31) than with climate indices across the last week (*n* = 25), or the previous week (*n* = 23), to the sampling date (Table 1).

In 19 stallions (61.3%) we observed no negative correlations between fertility rates of the respective weeks or the following weeks and the climate indices of the windows of 1 week and 2 weeks. These were deemed non-HISS stallions, and this group was used for further comparison of HISS and non-HISS stallion fertility, in terms of seminal and systemic biomarkers.

**Figure 3:**
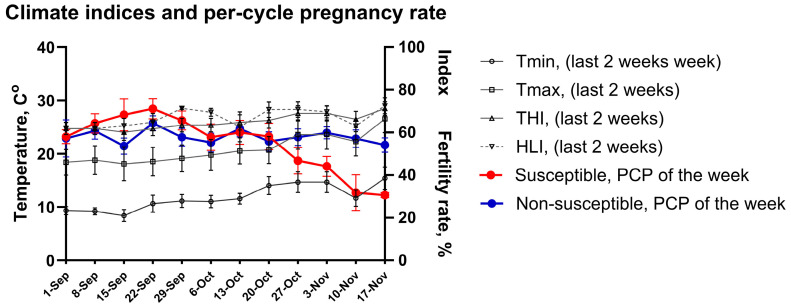
Week-to-week per-cycle fertility in HISS and non-HISS stallions. Per-cycle pregnancy (PCP) rates plotted alongside climate variables within preceding 2 weeks.

**Figure A1:**
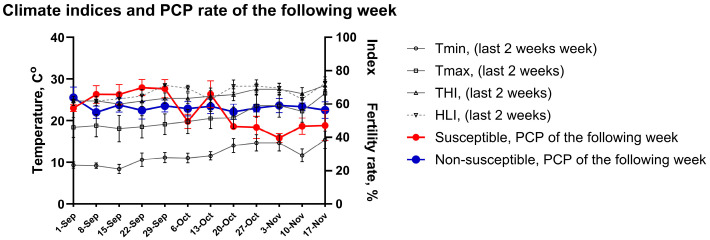
Per-cycle pregnancy (PCP) rates of the following week plotted alongside climate variables within the preceding 2 weeks in HISS and non-HISS stallions. Compared with the two-week window of climate and fertility rate, the three weeks window resulted in a slighter decrease in pregnancy rate in HISS stallions.

Weekly mean PCP rates for HISS and non-HISS stallions alongside the mean weekly climate parameters of the final two weeks are shown in Figure 3, with a distinct decrease in PCP observed in susceptible stallions. PCP for susceptible stallions decreased in the following week, with fluctuation, but not as severely as the initial week (Appendix A Figure A1).

### 3.2. Comparison of Sperm, Seminal and Systemic Parameters in HISS and Non-HISS Stallions

**Figure 4:**
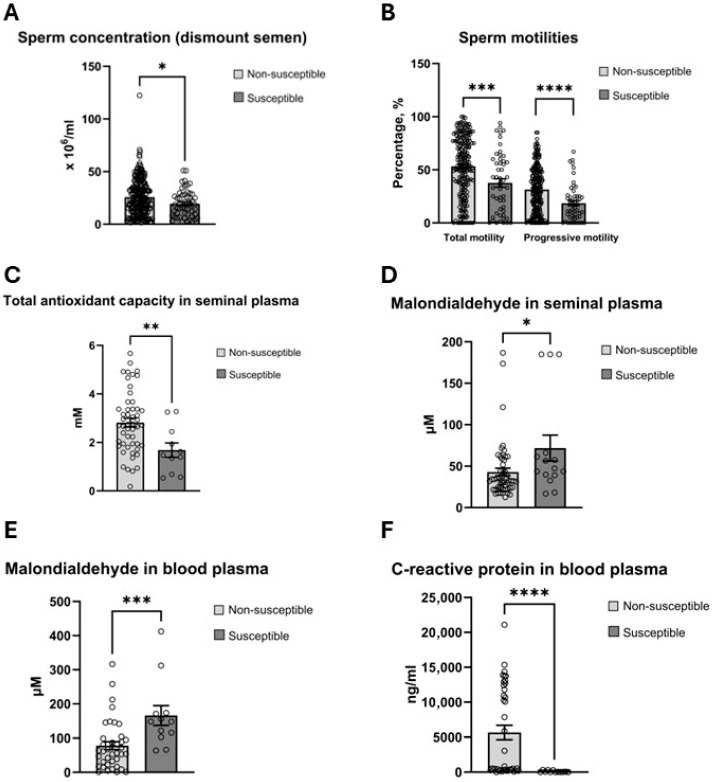
Significant differences in systemic and localized biomarkers were detected in HISS stallions. (**A**) Dismount semen sperm concentration was lower. (**B**) Total and progressive motility were low. (**C**) Seminal plasma TAC was low. (**D**) Seminal plasma MDA level was higher. (**E**) Systemic malondialdehyde was high. (**F**) Blood plasma CRP was 66 times lower in blood plasma. *, *p* < 0.05; **, *p* < 0.01; ***, *p* < 0.001; ****, *p* < 0.0001.

Markers of sperm quality (concentration, motility, DNA damage and chromatin compaction), seminal plasma redox measures (TAC, MDA), and circulating systemic markers of inflammation (CRP), blood plasma redox measures (TAC, MDA) and oxidative DNA damage (8-OH-2dG) in sperm DNA and blood plasma were compared between HISS and non-HISS stallions. HISS stallions presented lower sperm concentration (Figure 4A), noting these are residual dismount samples and may not be representative of total sperm output. Both total motility and progressive motility were significantly lower in HISS stallions (total motility: 37.65 ± 3.94% in HISS vs. 53.33 ± 1.95% in non-HISS; progressive motility: 18.61 ± 2.42% in susceptible vs. 31.39 ± 1.51% in non-susceptible; Figure 4B). HISS stallions showed significantly lower seminal plasma TAC (1.68 ± 0.29 mM vs. 2.82 ± 0.18 mM; Figure 4C) and higher MDA in seminal plasma (71.83 ± 15.53 µM vs. 42.99 ± 4.57 µM; Figure 4D) and blood plasma (165.85 ± 29.09 µM vs. 78.13 ± 11.55 µM; Figure 4E). Plasma CRP was extremely low in HISS stallions compared with non-HISS stallions (85.05 ± 33.71 ng/mL vs. 5652.82 ± 1023.03 ng/mL; Figure 4F).

**Figure 5:**
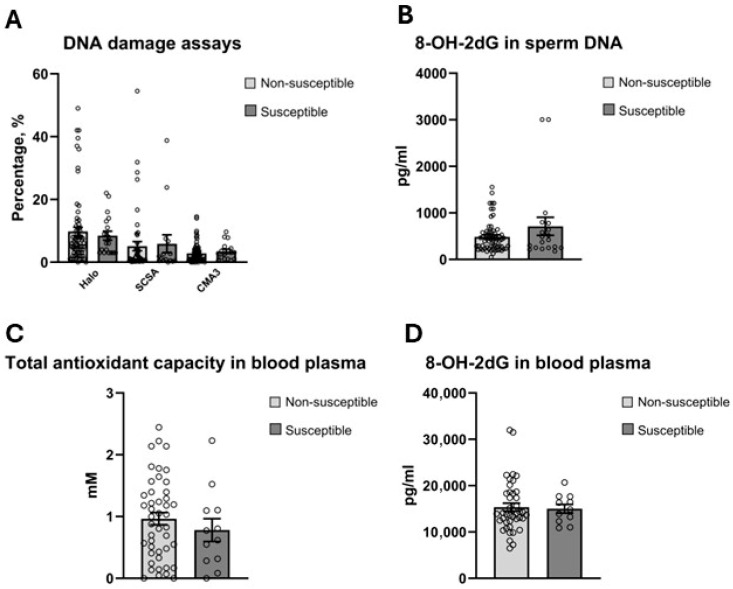
No significant difference detected in sperm DNA damage assays, oxidized deoxyguanine species in sperm DNA as well as in blood plasma and systemic total antioxidant capacity level. (**A**) Sperm chromatin dispersion assay (Halo), sperm chromatin structure assay (SCSA), and sperm chromatin maturity chromomycin A3 (CMA3) assays revealed no significant difference between the groups. (**B**) Oxidized deoxyguanosine in sperm DNA showed no difference. (**C**) Systemic total antioxidant capacity showed no difference, unlike in seminal plasma. (**D**) No difference was detected in 8-OH-2dG species in blood plasma.

Markers of sperm DNA damage and chromatin compaction, blood plasma TAC, and 8-OH-2dG in seminal plasma and blood plasma, did not differ significantly between HISS and non-HISS stallion groups (Figure 5).

### 3.3. Correlations of Sperm, Seminal and Systemic Parameters to Climate Variables in HISS and Non-HISS Stallions

**Figure 6:**
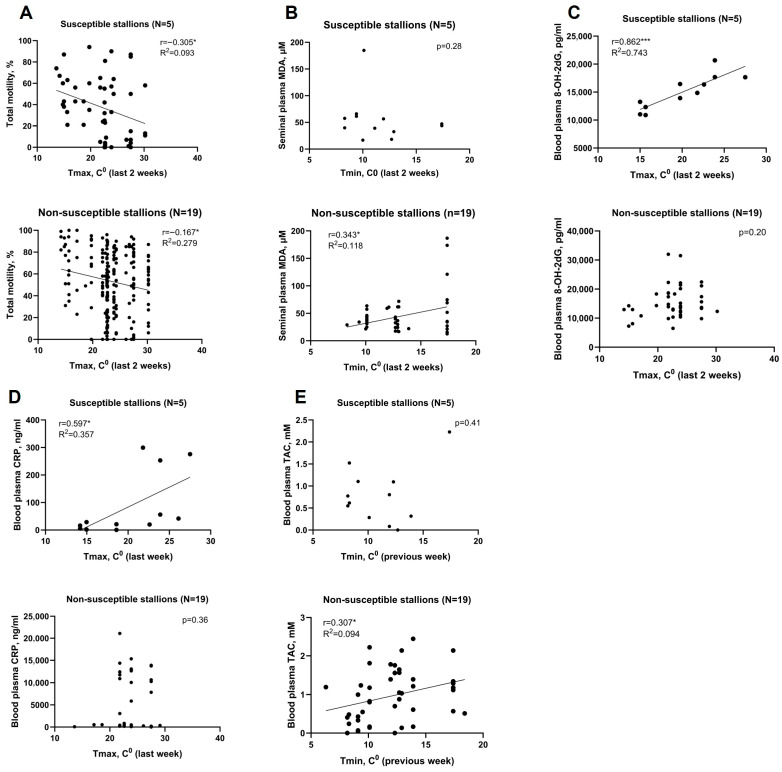
Biomarkers responded to climate indices differently between HISS and non-HISS stallions. (**A**) Total motility of HISS stallions responded inversely stronger to peak daytime temperature (highest correlation is shown). (**B**) Seminal plasma MDA responded moderately only in non-HISS stallions. (**C**) Blood plasma 8-OH-2dG level showing a very strong correlation to the peak temperature of daytime in HISS stallions. (**D**) CRP in HISS stallions had strong correlation to the peak temperature of the final week, not to other climate effect assessing periods. (**E**) Systemic TAC only responded to the lowest nighttime temperature of the initial week in non-HISS stallions. *, *p* < 0.05; ***, *p* < 0.001.

All sperm, seminal, and systemic markers were examined for correlation to climate indices to determine which measures might respond differentially to ambient heat. Total motility of HISS stallion sperm responded to Tmax more strongly (r = −0.305 *, R^2^ = 0.093) compared with non-HISS stallions (r = −0.167 *, R^2^ = 0.279; Figure 6A). Systemic 8-OH-2dG, and CRP of HISS stallions responded to Tmax strongly (r = 0.862 **, R^2^ = 0.743; r = 0.597 *, R^2^ = 0.375), but not in non-HISS stallions (Figure 6C,D). Conversely, systemic TAC and seminal plasma MDA responded to Tmax and Tmin (r = 0.307 *, R^2^ = 0.094; r = 0.343 *, R^2^ = 0.118) in non-HISS stallions, but these correlations were not observed in HISS stallions (Figure 6B,E).

### 3.4. Correlations Between Systemic Biomarkers, Sperm Quality and Stallion Fertility Rates

**Figure 7:**
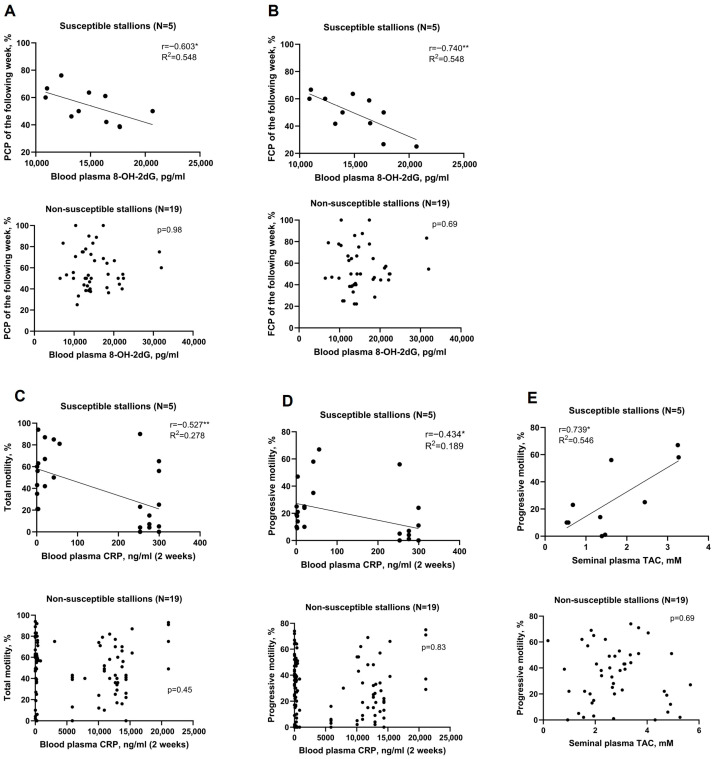
Seminal and systemic biomarker effects on fertility rates and sperm motility. (**A**,**B**) PCP rate of the following week and FCP rate of the following week had strong inverse correlation to systemic 8-OH-2dG in HISS stallions. (**C**,**D**) Systemic CRP had strong negative correlation to total motility and progressive motility in HISS stallions. (**E**) Seminal plasma antioxidant capacity had very strong correlation to progressive motility in HISS stallions. *, *p* < 0.05; **, *p* < 0.01.

Correlations between systemic markers of inflammation (e.g., CRP), redox status markers (MDA and TAC), oxidative DNA damage (8-OH-2dG), and reproductive measures (sperm quality and field pregnancy rates) were investigated and compared between HISS and non-HISS stallions. In HISS stallions, systemic 8-OH-2dG was inversely correlated to the PCP and FCP of the following week (r = −0.603 *, R^2^ = 0.548; r = −0.740 **, R^2^ = 0.548) (Figure 7A,B). Total and progressive motility both had negative correlation to CRP (r = −0.527 **, R^2^ = 0.189; r = −0.434 *, R^2^ = 0.189) (Figure 7C,D), but positive correlation to seminal plasma TAC (r = 0.739 *, R^2^ = 0.546) (Figure 7E). None of these correlations were observed in non-HISS stallions.

### 3.5. Interactions Between Systemic and Localized Reproductive Biomarkers

**Figure 8:**
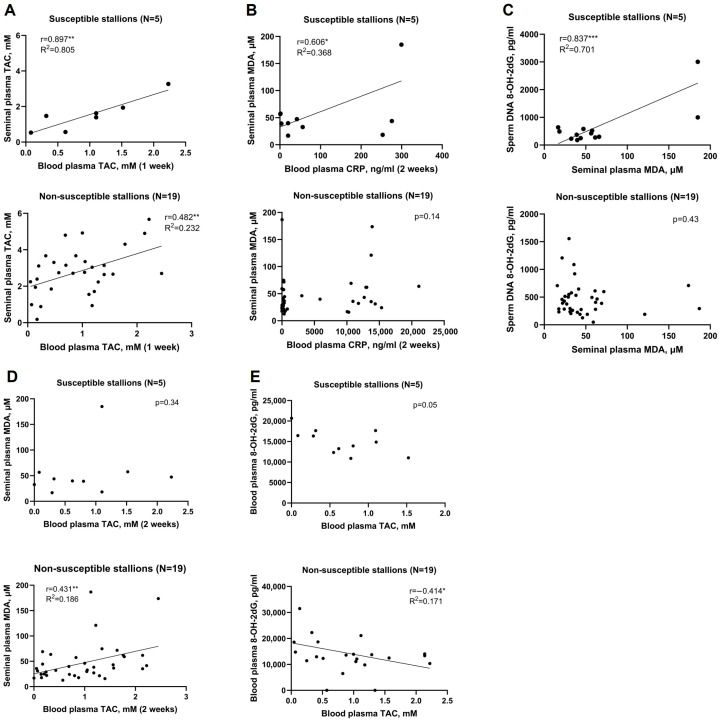
Systemic and localized biomarker interdependency. (**A**) Systemic, and seminal TAC had strong to very strong correlation in both groups. (**B**) Seminal plasma MDA were greatly influenced by blood plasma CRP in HISS stallions. (**C**) Seminal plasma MDA had very strongly correlation to oxidized guanine residue in sperm DNA. (**D**) Systemic TAC strongly influenced seminal MDA in non-HISS stallions, but not in HISS stallions. (**E**) Systemic oxidized guanine derivatives had strong correlation to systemic TAC in non-HISS stallions, not in HISS stallions. *, *p* < 0.05; **, *p* < 0.01; ***, *p* < 0.001.

Correlations of systemic and localized biomarkers revealed that seminal TAC is highly dependent on systemic TAC in HISS stallions (r = 0.897 **, R^2^ = 0.805) but less so in non-HISS stallions (r = 0.482 **, R^2^ = 0.232) (Figure 8A). HISS stallions exhibited strong correlations between CRP and seminal plasma MDA (r = 0.606 *, R^2^ = 0.368) (Figure 8B) and sperm DNA 8-OH-2dG was very strongly correlated to seminal plasma MDA (r = 0.837 ***, R^2^ = 0.701) (Figure 8C). On the other hand, in non-HISS stallions, seminal plasma MDA had moderate correlation to systemic antioxidant capacity (r = 0.431 **, R^2^ = 0.186) (Figure 8D) and moderate inverse correlation between systemic TAC and systemic 8-OH-2dG (r = −0.414 *, R^2^ = 0.171) (Figure 8E), which was not observed in HISS stallions.

## 4. Discussion

The link between ambient heat and reduced fertility is longstanding, yet the mechanism by which one leads to the other is not clearcut. Beyond direct effects on the testes, heat-induced subfertility may arise as the result of multiple heat-related physiological pathways with distinct phenotypes, including systemic heat stress, the heat shock response, or heat acclimation. Systemic heat stress is characterised by hyperthermia, inflammation and activation of the acute phase response [41]. The heat shock response is a cellular defence mechanism, characterised by cellular production of heat shock proteins (HSPs). These are deployed to mitigate cell and tissue damage and have a range of downstream signalling effects that may affect reproductive function [42,43]. Heat acclimation represents a longer-term physiological adaptation enabling survival under sustained thermal challenge and is mediated through chronic heat exposure and repeated activation of the heat shock response [44,45].

We observed that HISS stallions exhibit a distinct systemic phenotype, characterized by elevated oxidative stress and dramatically reduced acute phase protein response as measured by CRP. These stallions display significantly reduced seminal antioxidant capacity and heightened lipid peroxidation, in both seminal plasma and peripheral circulation. As this phenotype is consistent with that seen in heat-acclimated animals, we anticipate that subfertility in these stallions is a product of heat acclimation rather than a direct consequence of systemic heat stress.

Oxidative stress contributes to all three processes of heat stress, heat shock, and heat acclimation; however, the acclimated state is distinct in that organisms may exhibit an increased tolerance to oxidative load, with altered redox signalling and reduced activation of classical antioxidant and inflammatory responses [46]. While such a state is beneficial to survival and performance, the male reproductive system is exquisitely sensitive to oxidative stress [47], and as such, acclimation may be detrimental to fertility in hot conditions. These processes form a continuum from an acute, pro-inflammatory state (heat stress) to one of resilience and ability to withstand heat events with minimal tissue damage and attenuated inflammatory responses (heat acclimation) [48].

Given the profound potential of both oxidative stress and inflammatory processes to negatively impact male fertility, alongside their recognised roles in physiological responses to ambient heat, we hypothesised that antioxidant capacity and markers of end-products of oxidative stress (8-OH-2dG and MDA), and acute phase protein CRP may differentiate the HISS phenotype from stallions whose fertility is not observed to decrease following ambient heat events. We also sought to determine which systemic processes might be influencing reproductive parameters that could explain subfertility in these animals.

HISS stallions exhibited significantly reduced seminal antioxidant capacity alongside elevated lipid peroxidation in both seminal and blood plasma (Figure 4), indicating that these animals experience oxidative stress at both local and systemic levels. Importantly, seminal TAC in HISS stallions correlated tightly with systemic TAC, whereas non-susceptible stallions maintained greater independence between systemic and local antioxidant dynamics (Figure 8). This could suggest that susceptible animals are less able to recruit a localised antioxidant response independent of systemic levels.

Total and progressive sperm motility were lower in HISS stallions, and motility correlated strongly with seminal TAC (Figure 7E), supporting the idea of a role for local antioxidant defences in maintaining sperm function and fertility [49]. This is consistent with the experience of 19 non-HISS stallions who, in contrast to the HISS stallions, displayed weaker correlations between TAC and motility, suggesting antioxidant capacity may be a less impactful driver of sperm quality in this cohort. Seminal MDA correlated with sperm DNA oxidation—an expected finding but one that again supports a link between oxidative imbalance and functional sperm deficits in the HISS phenotype (Figure 8C).

Systemic oxidative DNA damage, as measured by 8-OH-2dG, was the only biomarker directly associated with fertility outcomes in HISS stallions, correlating negatively with per-cycle and first-cycle pregnancy rates (Figure 7A,B). Other studies have found similar outcomes [50,51,52,53]. Furthermore, systemic 8-OH-2dG had very strong correlation to peak day time temperature only in HISS stallions (Figure 6C). These relationships were not observed in non-HISS animals. We suspect that HISS stallions are less capable of shielding the reproductive system from the effects of systemic redox fluctuations. Together with the above findings, these results support the idea of a likely role for both systemic and localised oxidative stress in heat-associated subfertility.

A striking finding was the extremely low systemic CRP in HISS stallions, despite evidence of oxidative stress. CRP in these stallions responded to peak daytime temperatures (Figure 6D), yet its absolute levels remained dramatically lower (on average 66 fold lower) than in non-HISS animals, and consistently below the clinical reference range for normal healthy horses [54,55,56] suggesting that HISS stallions have a functioning, but altered, and tightly constrained acute phase response. An attenuated acute phase response has been observed in heat-acclimated animals [57,58,59]. CRP is induced by IL-6 which can in turn be regulated by heat shock proteins and testosterone [60,61].

It is plausible that the attenuated CRP response we observed in HISS stallions is either part of the heat shock response or the result of heat acclimation, and as such may represent a protective mechanism against excessive thermally induced inflammation. This could also explain the higher oxidative stress levels in HISS stallions, as an attenuated acute phase response can dampen the recruitment of antioxidants. While this would minimize tissue damage in the face of heat events and enhance the animal’s ability to withstand a higher level of oxidative stress, it may come at the cost of maintaining fertility. Further analysis of upstream regulators of the acute phase response, alongside measures of key heat shock proteins, will enable a deeper characterisation of this phenotype and of the question of whether subfertility indeed represents a byproduct of heat acclimation. Furthermore, if these features are associated with enhanced sporting performance under heat conditions, the consequent selective breeding decisions may have dire consequences for fertility in the longer term and as temperatures continue to increase.

This study relied on field data gathered from real-world ambient heat exposure of fertile stallions under commercial breeding conditions. As such, a limitation of the study is that the data are observational, limiting the extent to which causative, mechanistic links can be drawn between parameters measured and fertility outcomes. Nonetheless, these insights have both mechanistic and practical implications. Seminal TAC, seminal MDA, systemic MDA, and systemic CRP may serve as biomarkers for early identification of heat-susceptible stallions, enabling targeted management strategies such as antioxidant supplementation, modified breeding schedules, or environmental interventions to mitigate heat exposure. In future studies, exploring the molecular underpinnings of antioxidant mobilization and heat shock responses, including epididymal expression of antioxidant enzymes, accessory gland contributions, and heat shock protein activity, could reveal novel targets for enhancing reproductive resilience in heat-affected stallions.

## 5. Conclusions

This study demonstrates that ambient heat-associated subfertility in stallions is part of a distinct physiological phenotype more consistent with heat acclimation than acute systemic heat stress. Heat-induced subfertility-susceptible stallions exhibit alterations in systemic and local redox regulation, characterized by elevated oxidative stress, reduced antioxidant capacity within the reproductive tract, and a markedly attenuated acute phase response. Together, these findings suggest oxidative stress as a central mechanistic link between ambient heat exposure and impaired fertility, and underscore the importance of considering individual susceptibility when assessing reproductive risk under ambient heat conditions. As global temperatures continue to rise, further work to understand how adaptive heat responses intersect with reproductive function will be critical for safeguarding male fertility.

## Figures and Tables

**Table 1:** Correlation of ambient climate indices of different window periods and fertility rates of stallions.

Stallion Code	Climate Index	Last 7 Days	Last 2 Weeks	Previous Week
		r	r	r
2 (11 datapoints per correlation)	Tmin	−0.699 * (FCP of the week)	−0.874 *** (FCP of the week)	−0.699 * (FCP of the week)
		−0.768 ** (FCP of the following week)	−0.915 *** (FCP of the following week)	−0.768 ** (FCP of the following week)
			−0.714 * (PCP of the following week)	
			−0.638 * (PCP of the week)	
	Tmax	−0.858 *** (FCP of the week)	−0.891 *** (FCP of the week)	−0.858 *** (FCP of the week)
		−0.876 *** (FCP of the following week)	−0.921 *** (FCP of the following week)	−0.876 *** (FCP of the following week)
		−0.782 ** (PCP of the following week)	−0.808 ** (PCP of the following week)	−0.781 ** (PCP of the week)
		−0.781 ** (PCP of the week)	−0.858 *** (PCP of the week)	−0.782 ** (PCP of the following week)
	THI	−0.874 *** (FCP of the week)	−0.911 *** (FCP of the week)	−0.874 *** (FCP of the week)
		−0.855 *** (FCP of the following week)	−0.943 *** (FCP of the following week)	−0.855 *** (FCP of the following week)
		−0.703 * (PCP of the week)	−0.786 ** (PCP of the week)	−0.703 * (PCP of the week)
		−0.642 * (PCP of the following week)	−0.727 (PCP of the following week)	−0.642 * (PCP of the following week)
	HLI		−0.620 * (FCP of the week)	
			−0.758 * (FCP of the following week)	
12 (10 data points per correlation)	Tmin		−0.645 * (PCP of the week)	
			−0.786 ** (PCP of the following week)	
	Tmax	−0.859 ** (PCP of the week)	−0.819 ** (PCP of the week)	−0.638 * (PCP of the week)
		−0.703 * (PCP of the following week)	−0.780 ** (PCP of the following week)	−0.888 *** (PCP of the following week)
	THI	−0.755 * (PCP of the week)	−0.756 * (PCP of the week)	−0.669 * (PCP of the week)
		−0.675 * (PCP of the following week)		−0.901 *** (PCP of the following week)
	HLI			−0.669 *** (FCP of the following week)
21 (10 data points per correlation)	Tmin		−0.636 * (PCP of the week)	
	Tmax		−0.636 * (PCP of the week)	−0.649 * (PCP of the week)
				−0.682 * (PCP of the following week)
	THI		−0.647 * (PCP of the week)	
	HLI	−0.689 * (FCP of the week)		
		−0.718 * (FCP of the following week)		
		−0.646 * (PCP of the week)		
		−0.646 * (PCP of the following week)		
24 (11 data points per correlation)	Tmin	−0.690 * (FCP of the week)	−0.727 * (FCP of the week)	−0.603 * (PCP of the following week)
			−0.714 * (PCP of the week)	
	Tmax	−0.758 ** (FCP of the week)	−0.774 ** (FCP of the week)	−0.642 * (FCP of the week)
		−0.778 ** (PCP of the week)	−0.770 ** (PCP of the week)	−0.694 * (PCP of the week)
	THI	−0.743 ** (FCP of the week)	−0.774 ** (FCP of the week)	−0.699 * (PCP of the week)
		−0647 * (PCP of the week)	−0.800 ** (PCP of the week)	
	HLI		−0.662 * (PCP of the following week)	
27 (10 data points per correlation)	Tmax	−0.659 * (PCP of the following week)	−0.720 * (PCP of the following week)	−0.661 * (PCP of the week)
				−0.711 * (PCP of the following week)
	THI	−0.644 * (PCP of the following week)	−0.683 * (PCP of the following week)	

PCP: per cycle pregnancy; FCP: first cycle pregnancy; THI: temperature–humidity index; HLI: heat load index; Tmin: lowest nighttime temperature; Tmax: daytime peak temperature. *, *p* < 0.05; **, *p* < 0.01; ***, *p* < 0.001.

## Data Availability

Climate data utilized in this study are available at the Bureau of Meteorology (https://reg.bom.gov.au/climate/data/). Apart from datasets included in the article, all other data generated in this study are available from the corresponding author upon request.

## Abbreviations

Tmax	Daytime peak temperature
Tmin	Nighttime peak temperature
HLI	Heat load index
THI	Temperature–humidity index
TAC	Total antioxidant capacity
MDA	Malondialdehyde
8-OH-2dG	Oxidized guanine species
ELISA	Enzyme-linked immunosorbent assay
SCSA	Sperm chromatin structure assay
CMA3	Chromomycin A3
CRP	C-reactive protein
HISS	Heat-induced subfertility susceptible
DNA	Deoxyribonucleic acid
FCP	First cycle pregnancy
PCP	Per-cycle pregnancy
RT	Room temperature
EDTA	Ethylenediaminetetraacetic acid
HEPES	N-2-hydroxyethylpiperazine-N′-2-ethanesulfonic acid
BWW	Biggers, Whitten and Whittingham
VAP	Velocity of average path
VCL	Velocity of curvilinear
VSL	Velocity of straight line
STR	Straightness
TBR	Tris–boric acid–EDTA
DAPI	4′,6-diamidino-2-phenylindole
